# Toxicity Assessment of Carbon Nanomaterials in Zebrafish during Development

**DOI:** 10.3390/nano7120414

**Published:** 2017-11-25

**Authors:** Marta d’Amora, Adalberto Camisasca, Stefania Lettieri, Silvia Giordani

**Affiliations:** 1 Nano Carbon Materials, Istituto Italiano di Tecnologia (IIT), via Livorno 60, 10144 Torino, Italy; marta.damora@iit.it (M.d.A.); adalberto.camisasca@iit.it (A.C.); stefania.lettieri@iit.it (S.L.); 2 Department of Chemistry and Industrial Chemistry, University of Genoa, via Dodecaneso 31, 16145 Genoa, Italy; 3 Department of Chemistry, University of Turin, via Giuria 7, 10125 Turin, Italy

**Keywords:** nanotoxicology, carbon nanomaterial, zebrafish, carbon nano-onion, carbon nano-horn, graphene oxide

## Abstract

Carbon nanomaterials (CNMs) are increasingly employed in nanomedicine as carriers for intracellular transport of drugs, imaging probes, and therapeutics agents, thanks to their unique optical and physicochemical properties. However, a better understanding about the effects of CNMs on a vertebrate model at the whole animal level is required. In this study, we compare the toxicity of oxidized carbon nano-onions (oxi-CNOs), oxidized carbon nano-horns (oxi-CNHs) and graphene oxide (GO) in zebrafish (*Danio rerio*). We evaluate the possible effects of these nanomaterials on zebrafish development by assessing different end-points and exposure periods.

## 1. Introduction

Since the discovery of fullerene (C_60_) by Kroto et al. in 1985 [1], carbon nanomaterials (CNMs) have gained increased interest in different disciplines, spanning from electronic to biomedical applications. In the past years, several new CNMs have been discovered, namely, carbon nanotubes (CNTs) [2], carbon nano-horns (CNHs) [3], carbon nano-onions (CNOs) [4], graphene [5,6] and nanodiamonds (NDs) [7].

They are promising materials for intracellular transport of drugs, as imaging probes and as therapeutics agents, thanks to their chemico-physical characteristics and their nanometer size. GO was first discovered in 1859 via harsh oxidative treatment of graphite [8] and then modified via the Hummers method [9]. GO and nano-graphene oxide (NGO) are used for imaging, therapeutics and diagnostic applications [10,11]. Previous works have investigated the in vitro toxicity of GO on different cell lines, including lung cancer cells [12], skin keratinocytes [13], human umbilical vein endothelial cells (HUVECs) [14], and human fibroblast cells [15]. GO induced cytotoxicity and genotoxicity with a size- and dose-dependent behavior, leading to oxidative stress and apoptosis. In addition, different studies reported the non-toxicity or low toxicity of graphene oxide in zebrafish during development, showing that embryos exposed or injected with GO presented an inhibition of the hatching rate and an increase of morphological abnormalities [16,17,18,19]. Moreover, one of the drawback of graphene and GO was the in vivo toxicity towards lung [20]. In particular, their toxicity was related to the dimension of the flakes lateral size, with larger flakes being more toxic than the smaller one [21].

CNHs are conical carbon nanostructures constructed from a closed cages of sp^2^-bonded carbon atoms, typically 2−5 nm in diameter and 40−50 nm in length [19]. CNHs can aggregate to form dahlia or bud-like structures with an overall diameter of 100 nm. They are promising nanostructures for biological applications, e.g. drug carriers [22,23]. However, they can cause cell toxicity, due to their tendency to produce reactive oxygen species [24]. In addition, in vivo toxicity of CHNs was evaluated in mice only with specific tests, reporting non-toxic effects of this nanomaterial on the skin and eyes [25]. A complete in vivo toxicological assessment is still needed. Carbon nano-onions (CNOs) are multi-shell fullerenes [4,26] discovered by Iijima in 1980 [27] and later reported by Ugarte, which observed the in situ formation of onion-like graphitic nanoparticles from amorphous carbon [4]. CNOs are an attractive nanomaterials for imaging, diagnostic and therapeutic applications, due to their small size and spherical shape [28]. Our recent reports showed that surface functionalized CNOs presented low inflammatory potential [29], weak cytotoxicity in vitro, on different cell lines, including human breast adenocarcinoma cells (MCF-7 cells) [30,31], HeLa cells [32,33,34,35], HeLa Kyoto cells [36] and human cervix carcinoma cells, derivative of HeLa (KB cells) [33] and they were uptaken by cancer cells and accumulated in the lysosomes [31,33,35]. In addition, we probed that CNOs did not exert toxic effects in *Hydra vulgari* [37] and had high biocompatibility in zebrafish during the development [38].

Here, we compare the toxicity of carbon nano-onions (CNOs), carbon nanohorns (CNHs) and graphene oxide (GO) in zebrafish, to understand and evaluate which CNMs is potentially more adequate for biological applications. Although several reports have demonstrated the potentiality of GO, CNHs and CNOs for biological applications, there is still the need to perform more studies on their effects on a more complex and vertebrate system. Doubt on the validity of some well-established rodent disease models question the use of these models to screen therapeutic agents on a live animal, as the obtained results can be misleading and potentially costly if a drug or drug delivery system does not behave in the same way on humans. Although a more comprehensive elucidation of the resemblances and differences between zebrafish and human biology is still needed, the use of zebrafish as a vertebrate model is potentially ideal as it has a high degree of physiology and metabolism conservation with humans [39]. Moreover, zebrafish embryos exhibit optical transparency, external development and short breeding cycle, which allow the use of this model for high-throughput screening [40,41]. Previous studies on the biocompatibility of other CNMs in zebrafish during development reported a dose and time-dependent toxicity, high mortality rate, and different embryos/larvae malformations. In particular, fullerenes [42,43], single-walled carbon nanotubes (SWCNTs) and graphene quantum dots [44] induced developmental delay and different malformations. Only carbon quantum dots (C-QDs) were found to be biocompatible and exhibited low toxicity in zebrafish [44]. Herein we wanted to compare three different CNMs, to better understand their effect on a live animal, and fully establish which one can be the best candidate for biological applications.

For these studies, we oxidized both CNOs (oxi-CNOs) and CNHs (oxi-CNHs), to chemically introduce carboxylic groups on their surface, as the presence of -COOH functional groups enhances their solubility in aqueous media and increases their biocompatibility, as previously demonstrated also for carbon nanotubes [30,45]. In addition, we tested a commercially available GO. The herein described work reported on the evaluation of different toxicological end-points on zebrafish embryos and larvae treated with oxi-CNOs, oxi-CNHs and GO.

## 2. Results and Discussion

### 2.1 Preparation and Characterization of CNMs

**Figure 1:**
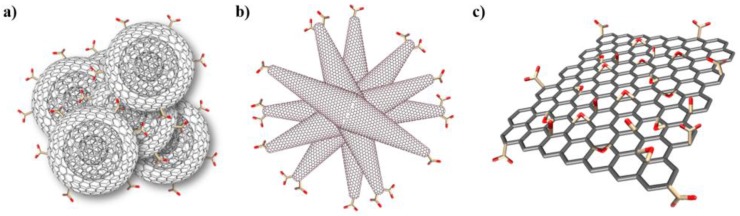
3D representation of oxidized carbon nanomaterials (CNMs): (**a**) Oxidized carbon nano-onions (oxi-CNOs); (**b**) oxidized carbon nano-horns (oxi-CNHs) and (**c**) graphene oxide (GO).

We investigated the effects induced by three different oxidized CNMs, schematically reported in Figure 1, on zebrafish during development. The surface of pristine CNOs (p-CNOs) and pristine CNHs (p-CNHs) were decorated with carboxylic acid functionalities, following previously reported procedures [32,45], while GO was used as received.

In order to confirm the successful covalent functionalization, pristine and oxidized carbon nanomaterials (CNMs) were characterized by means of different techniques, such as thermogravimetric analysis (TGA), Raman and X-ray photoelectron spectroscopies (XPS) and transmission electron microscopy (TEM).

Raman spectroscopy is a versatile and non-destructive tool for structural characterization of carbon-based materials. The Raman spectra of the different oxidized nano-materials are shown in Figure S1b, and are characterized by two main peaks: the G-band (in the range 1320–1340 cm^−1^), which is assigned to the E_2g_ phonon mode at the Brillouin zone center, originating from in-plane vibration of sp^2^ hybridized carbon, and the D-band (in the range 1580–1600 cm^−1^), which is a defect-induced Raman feature arising from breathing mode of photons of A_1g_ symmetry [46]. Raman spectra of oxi-CNOs and oxi-CNHs display two additional features with weaker intensity in the range 2500–3000 cm^−1^. The 2D-band at around 2650 cm^−1^ arises from a two-phonon resonant scattering process and refers to the overtone of the D-band, while the band at about 2950 cm^−1^ corresponds to the combinational scattering (D + G combination mode) [47]. By contrary, the Raman spectrum of GO displays a highly broadened and low intensity 2D region. The relative strength of the D-band compared to G-band (I_D_/I_G_ ratio) depends strongly on the disorder present in the graphitic materials [48]. Upon oxidation, the D-band of oxidized materials becomes more prominent compared to the pristine materials (Figure S1a), resulting in the increase of I_D_/I_G_ ratio, which suggests structural changes due to the introduction of carboxylic acid groups upon oxidation. Table S1 summarizes the different values for each sample.

The thermal stability of the samples was evaluated by means of TGA, confirming their successful functionalization. Both pristine CNOs and CNHs (Figure S2a) exhibited high thermal stability, with negligible weight loss until their complete decomposition with the main mass loss observed at 686 and 665 °C, respectively. The TGA plots of oxi-CNOs, oxi-CNHs and GO, under air atmosphere, are shown in Figure S2b. After the oxidation, oxi-CNOs and oxi-CNHs reveal a decreased decomposition temperature (667 and 649 °C, respectively) and a net weight loss, due to the thermal decomposition of the COOH functionalities, followed by the decomposition of the carbon core. The TGA weight loss of the pristine sample compared to the oxidized one revealed a mass loss at 450 °C equal to 7.8% for oxi-CNOs and 10.87% for oxi-CNHs. Contrarily, GO displays a different behavior compared to the other CNMs; below 100 °C, an initial mass loss (about 11 wt. %) is observed due to the loss of water adsorbed on the interlayer spaces of GO sheets due to its hydrophilic nature. Above 100 °C, GO display a two-step decomposition process with two significant drops in mass around 200 and 450 °C. The first stage is attributed to the decomposition of the oxygen-containing functionalities, yielding CO and CO_2_, with a weight loss of about 30%, while the second to the pyrolysis of GO carbon skeleton. Table S2 summarizes the decomposition temperature for the different CNMs.

XPS was performed to investigate the surface composition and the degree of oxidation of the different CNMs, before and after the oxidation. The XPS survey spectra of pristine CNOs and CNHs are shown in Figure S3. For both pristine materials, the sample is mainly composed of carbon and the initial oxygen concentration is very low, while, after the oxidation, the oxygen content greatly increases in both samples (Figure S4). XPS survey spectrum of GO (Figure S4c) exhibits a considerable degree of oxidation; dominant carbon and oxygen features (69.73% and 27.66%, respectively) are observed along with traces of residual nitrogen and sulfur left over from the synthesis of graphene oxide. Table S3 reports the atomic percentages of all the samples.

**Figure 2:**
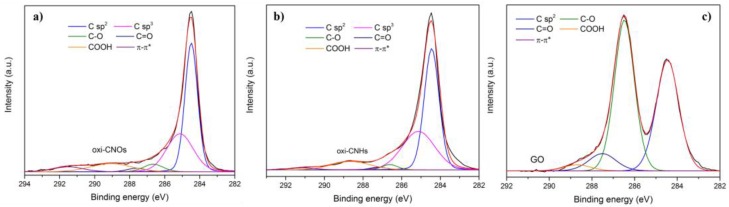
XPS spectra of the C1s region of (**a**) oxi-CNOs; (**b**) oxi-CNHs and (**c**) GO, including peak-fitting analysis.

To evaluate the functional groups of each sample, high-resolution XPS C1s spectra were acquired. All spectra were calibrated to the C1s binding energy (284.5 eV) of the graphitic peak and the corresponding curve fitting was made with a Gaussian-Lorentzian peak shape after performing a Shirley background correction; the position and the relative area percentages of the different C1s peaks are summarized in Table S4. The C1s XPS spectra of pristine CNOs and CNHs (Figure S5) were deconvoluted into five individual peaks, corresponding to the carbon atoms of different functional groups. Characteristic peaks at around 284.5, 285.5, 286.5, 288 and 291 eV were attributed to graphitic carbon (C–C), carbon atoms with sp^3^ hybridization, hydroxyl and epoxy/ether groups (C–O), carbonyl/quinone groups (C=O) and a satellite peak corresponding to the π-π* transition in the aromatic systems, respectively. After the surface modification, a new peak at around 289 eV was observed in the C1s spectra of oxidized CNMs (Figure 2), corresponding to carboxyl groups (COOH) introduced by the oxidation procedure.

Figure 2c shows the high resolution XPS C1s spectrum of GO, which was deconvoluted into four different peaks. The graphitic peak is centered at 284.45 eV, while the peaks at 286.48, 287.51 and 288.69 eV were assigned to C–O, C=O and COOH groups; furthermore, the vanishing of the π-π* shake up satellite observed is due to the disappearance of un-oxidized aromatic domains over the carbon network.

**Figure 3:**
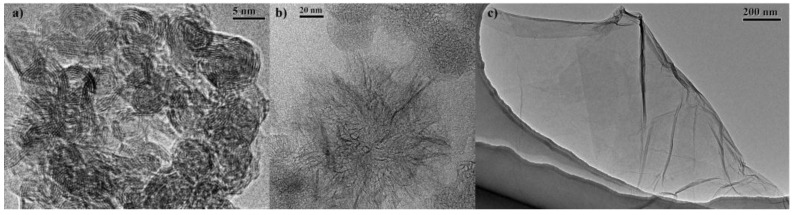
HRTEM images of (**a**) oxi-CNOs; (**b**) oxi-CNHs and (**c**) bright field TEM image of GO.

The structural and morphological properties were evaluated by high-resolution transmission electron microscopy (HRTEM), to confirm the integrity of the different structures after the oxidation procedure. HRTEM images of p-CNOs display the typical concentric structure [26] consisting of 6–8 graphitic shells 3.4 Å apart and an average diameter of 5–8 nm (Figure S6a). p-CNHs are composed of two different kind of CNHs aggregates, dahlia- and bud-like, depending if the graphene sheets protrude or not from the surface of the aggregate, and exhibit an average diameter of 80–100 nm [49,50] (Figure S6b). After the oxidation, no changes in the structure of both samples were observed, as suggested by HRTEM images (Figure 3). Characteristic bright field TEM image of GO is shown in Figure 3c, revealing the presence of few-layered GO sheets composed of a transparent and thin wrinkled paper-like structure.

### 2.2 Embryonic Toxicity

To assess and compare the possible effects of oxi-CNOs, oxi-CNHs and GO on zebrafish during the development, embryos were treated with different concentrations (5, 10, 50, 100 μg·mL^−1^) of these CNMs for 120 hours post fertilization (hpf). Different toxicological endpoints were investigated during a continuous period, at different stages of development (4, 24, 48, 72, 96, 120 hpf).

**Figure 4:**
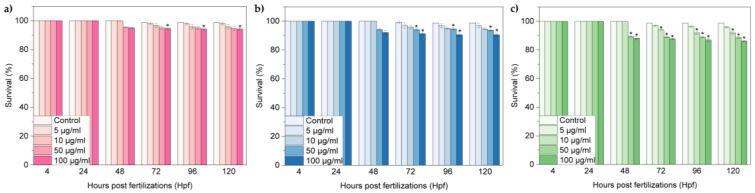
Survival rate of zebrafish treated with (**a**) oxi-CNOs, (**b**) oxi-CNHs and (**c**) GO. Values are expressed as means ±S.D.

Figure 4a–c shows the survival rates of zebrafish embryos exposed to oxi-CNOs, oxi-CNHs and GO respectively. At lower concentrations of oxi-CNOs and oxi-CNHs, no differences in the survival rates of treated samples were observed respect to the control; significant differences (p ≤ 0.01) were observed when increasing the dosages of CNOs (100 μg·mL^−1^) and CNHs (50 and 100 μg·mL^−1^) between 72 and 120 hpf (Figure 4a,b). In the case of GO, the survival rate exhibited a time and concentration-dependent behavior, comparable with the control group only at 5 μg·mL^−1^ between 72 and 120 hpf (Figure 4c). At higher concentration, the survival rate decreased significantly between 72 and 120 hpf. In particular, at 50 and 100 μg·mL^−1^, the survival rates were under 90% in this temporal window.

**Figure 5:**
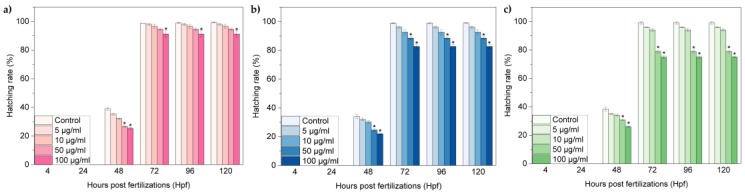
Hatching rates of zebrafish treated with (**a**) oxi-CNOs, (**b**) oxi-CNHs and (**c**) GO. Values are expressed as means ±S.D.

The hatch normally occurs between 48 and 72 hpf and it is as a critical stage for zebrafish embryogenesis. Figure 5a–c show the hatching rates of zebrafish embryos exposed to oxi-CNOs, oxi-CNHs and GO, respectively. The hatching rates of the three carbon nanomaterials exhibited a time and concentration-dependent behaviour. In particular, for the CNOs and CNHs, until 10 μg·mL^−1^, embryos hatched normally between 48 and 72 hpf (Figure 5a,b). At 100 μg·mL^−1^ of CNOs, and 50 and 100 μg·mL^−1^ of CNHs, the hatching rates were significantly lower (p ≤ 0.01), in comparison to the control samples. In contrast, GO induced a significant reduction of the hatching rates, with developmental delay (Figure 5c). At 50 and 100 μg·mL^−1^, the survival rates were under 80% between 72 and 120 hpf, respectively.

**Figure 6:**
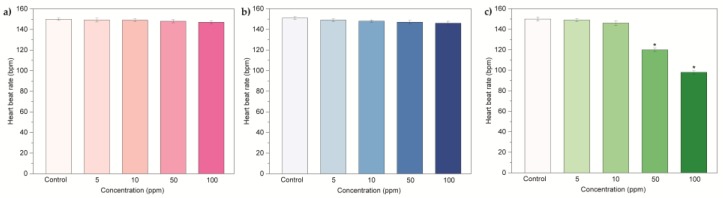
Heart beat rate of zebrafish treated with (**a**) oxi-CNOs, (**b**) oxi-CNHs and (**c**) GO. Values are expressed as means ±S.D.

The value of survival and hatching rates indicated different biological consequences induced by oxi- CNOs, oxi-CNHs and GO. In accordance with the OECD guidelines [51], to assume as non-toxic a nanomaterial, the percentage values of survival should be ≥ 90%, while the values of hatching should be ≥80%. Therefore, our results indicated that CNOs and CNHs did not exerted toxic effects in zebrafish for all the tested concentration. In contrast, GO induced toxicity with consequent developmental delay on zebrafish during development at high concentration (50 and 100 μg·mL^−1^). Next, we evaluated the behavioral toxicological endpoints in terms of two different parameters, the heart beat rate and frequency of movements. The parameters were observed in larvae at 72 and 96 hpf, respectively. The heart rates of larvae exposed to oxi-CNOs and oxi-CNHs were comparable with the control samples (Figure 6a,b). In the embryos treated with GO, the heart beats were significantly (p ≤ 0.01) reduced at 50 and 100 μg·mL^−1^ (Figure 6c).

**Figure 7:**
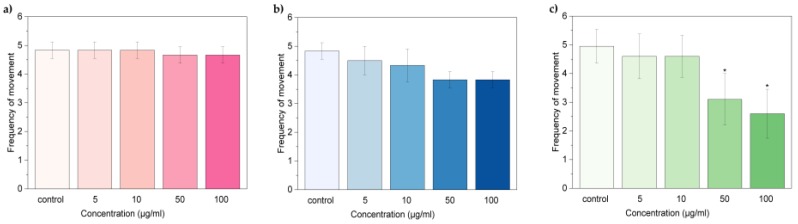
Frequency of movement of zebrafish larvae exposed to (**a**) oxi-CNOs, (**b**) CNHs and (**c**) GO. Values are expressed as means ±S.D

A similar trend was observed also for the frequency of movement (Figure 7a–c), with no significant reduction for larvae treated with oxi-CNOs and oxi-CNHs and notable reduction for larvae treated with 50 and 100 μg·mL^−1^ of GO.

These data indicated that oxi-CNOs and oxi-CNHs had no effects on the heart beat rate and frequency of movement of zebrafish, while these parameters were affected by GO.

**Figure 8:**
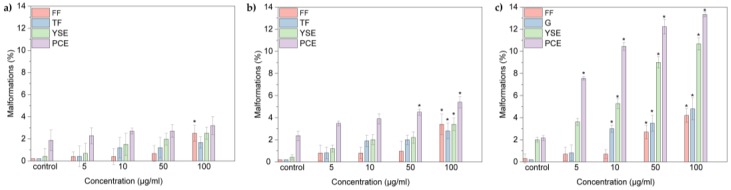
Malformation of larvae at 96 hpf treated with (**a**) oxi-CNOs, (**b**) oxi-CNHs and (**c**) GO. FF, fin fold flexure; TF, tail flexure; YSE, yolk sac edema; PCE, pericardial edema.

Also, the possible developmental defects induced by oxi-CNOs, oxi-CNHs and GO on zebrafish larvae were evaluated (Figure 8a–c).

**Figure 9:**
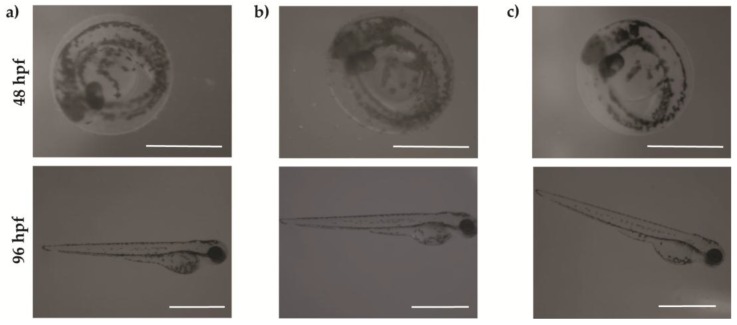
Optical images of embryos at 48 hpf and larvae at 96 hpf treated with 100 μg·mL^−1^ of (**a**) oxi-CNOs; (**b**) oxi-CNHs and (**c**) GO. Scale bar = 1 mm.

**Figure 10:**
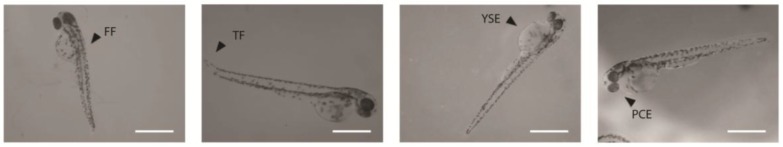
Representative optical images of the four different malformations observed in larvae at 96 hpf treated with oxi-CNOs or oxi-CNHs or GO. FF, fin fold flexure; TF, tail flexure; YSE, yolk sac edema; PCE, pericardial edema. Scale bar = 1 mm.

Figure 9 shows that embryos and larvae treated with the higher concentration of CNOs, CNHs and GO grow normally. We observed four types of malformations: Fin fold flexure (FF), tail flexure (TF), yolk sac edema (YSE), and pericardial edema (PCE) (Figure 8 and Figure 10). Oxidized CNOs and CNHs induced very low percentage of abnormalities, under 5% at the higher concentration. These low values indicated that both materials did not exerted toxicity on the zebrafish organogenesis. On the other hand, GO induced relevant percentage of malformations, indicating their toxicity.

Until 72 hpf, as shown in the optical images (Figure 9), the embryos are surrounded by a chorion which is as a barrier against the surrounding environment in early life-stages. It presents pore canals of approximately 0.6 mm in diameter, allowing the nanomaterials to pass through the chorion by passive diffusion. Zebrafish embryos presents a transparent chorion surface, without visible black spots on it. This indicates that the three nanomaterials did not appear to agglomerate in large clusters, and did not block the chorion pores.

In conclusion, we compared the toxicity of oxi-CNOs, oxi-CNHs and GO using a five-day embryonic zebrafish assay. Our results clearly demonstrated the biosafety of oxi-CNOs and oxi-CNHs at the concentrations evaluated and the toxicity of GO s in a dose-dependent manner on zebrafish with regard to hatching and development. Above 50 μg·mL^−1^, embryos and larvae treated with GO presented a survival rate under 85%, hatching rate/time disturbance, a developmental delay with different malformations and a decrease of spontaneous movements. Moreover, comparing the different values related to the toxicological endpoints of CNOs and CNHs, we can conclude that CNOs presented a higher biocompatibility than CNHs, with a difference of 5–10 in percentage for the different parameters, showing the most promising features for biological applications.

The different toxicological behavior of the three carbon nanomaterials depends on their physio-chemical properties. The three CNMs, as mentioned before, have different shapes, average size and surface areas affecting their interaction with biological systems. Oxi-CNOs are the nanoparticles with higher biocompatibility, which can be ascribed to their spherical shape in comparison to the other carbon nanomaterials investigated in this work, which exhibit sharper edges. Although oxi-CNHs still exhibited a good biocompatibility, their toxicity is slightly higher than oxi-CNOs, probably caused by their nanometer-sized graphitic conical structure. Moreover, from literature CNHs showed a small toxicity in cells, due to their tendency to produce reactive oxygen species. Above all CNMs here reported, GO was the most toxic. GO cytotoxicity is size-dependent, hence we can assume that, their lateral size which is not at the nanometer size, can play an important role in its biocompatibility.

## 3. Materials and Methods

### 3.1. Nanomaterials Synthesis

All reagents and solvents were purchased from Sigma-Aldrich and were used as received. Detonation nano-diamonds (DNDs) (uDiamond®Molto) were purchased from Carbodeon Ltd., pristine single-walled carbon nano-horns (p-CNHs) were purchased from Carbonium srl and graphene oxide (GO) was purchased from Graphenea Inc.; all nanomaterials were used as received with no further purification.

#### 3.1.1. p-CNOs

The synthesis of 5 nm p-CNOs was accomplished by thermal annealing of DNDs of 4–6 nm average particle size in a furnace in helium at 1650 °C followed by an air annealing at 450 °C to remove amorphous carbon [52].

#### 3.1.2. oxi-CNOs

The oxidation of p-CNOs was performed following the procedure reported in ref [32]. Briefly, 50 mg of p-CNOs were dispersed in 30 mL of 3 M nitric acid (HNO_3_) by ultrasonication (20’ at 37 kHz) and stirred for 48 h. The oxi-CNOs were firstly centrifugated (15’ at 1800 rpm) to remove the excess of nitric acid and then filtered and washed with dH_2_O, DMF, methanol and acetone on a nylon filter membrane (pore size 0.2 μm) to recover 52 mg of oxi-CNOs.

#### 3.1.3. oxi-CNHs

The oxidation of p-CNHs was performed following the procedure reported in ref [45]. Briefly, a dispersion of p-CNHs (10 mg) was prepared by ultrasonication (10’ at 37 kHz) in a 7M HNO_3_ solution. The solution was heated at 110 °C for 30’, and then cooled down in ice. The oxi-CNHs were separated from the nitric acid solution by centrifugation (10’ at 1200 rpm) and purified by several redispersion-centrifugation steps in dH_2_O and ethanol (10’ at 1200 rpm). After drying, 15 mg of oxi-CNHs were recovered.

### 3.2. Nanomaterials Characterization

#### 3.2.1. Thermogravimetric Analysis (TGA)

TGA was performed on a TA Q500 analyzer (TA Instruments, New Castle, PA, USA), using a Pt pan as sample holder. The measurement was performed in air using a heating rate of 10 °C/min.

#### 3.2.2. Raman Spectroscopy

Raman spectra were acquired on a Horiba Jobin Yvon HR 800 UV (Horiba, Kyoto, Japan) LabRam Raman microscope depositing. For the Raman measurements, the samples were deposited on a silicon wafer using an excitation wavelength of 632 nm.

#### 3.2.3. Transmission Electron Microscopy (TEM)

Bright-field TEM imaging of GO was performed on a Jeol JEM-1011 instrument (Jeol, Tokyo, Japan) equipped with a thermoionic tungsten source operated at 100 kV. High-resolution TEM (HRTEM) images of pristine and oxidized CNOs were recorded with a Jeol JEM-2100 instrument (Jeol, Tokyo, Japan) operated at 80 kV. HRTEM observations of pristine and oxidized CNHs were carried out with a Jeol 3010 instrument (Jeol, Tokyo, Japan) operated at 300 kV. TEM grids were prepared by spreading a droplet of the dispersed particles in ethanol on a copper grid coated with a lacey carbon film.

#### 3.2.4. X-ray Photoelectron Spectroscopy (XPS)

XPS analyses were carried out using an X-ray photoelectron spectroscopy microprobe (PHI Quantes, ULVAC-PHI) (Kanagawa, Japan) with a monochromatic Al Kα (1486.6 eV) radiation source. The specimens were prepared dropping a droplet of solution of the samples dispersed in ethanol onto an Au coated Si substrate so that the surface charge caused by generated photoelectrons could be dramatically reduced. Fitting procedure and deconvolution analyses were performed by using Multipak 9.6 software. The core level peak energies were calibrated to the C1s peak at 284.5 eV (C–C/C–H sp^2^ bonds). The peak listed as * in the XPS survey spectra of p- and oxi-CNOs were assigned to the Au substrate used for the analysis.

### 3.3. In Vivo Toxicity Studies

#### 3.3.1. Zebrafish Maintenance

Adult zebrafish were maintained as previously described [38].

#### 3.3.2. Zebrafish Embryo Exposure to oxi-CNOs, CNHs and GO

At 4hpf healthy embryos were transferred into 24-well culture plates with embryo medium (NaCl 5.03 mM, KCl 0.17 mM, CaCl_2_ 0.33 mM, and MgSO_4_ 0.33 mM) (2 embryos in 1 mL of medium per well; 80 embryos in total). Oxi-CNOs, oxi-CNHs and GO dispersions (5, 10, 50, 100 μg·mL^−1^) were dissolved in embryo medium and sonicated for 10 min. Eggs were treated with different concentrations of oxi-CNOs, oxi-CNHs and GO (5, 10, 50, 100 μg·mL^−1^) at 28 °C for 120 hpf. Embryo medium was used as negative control. The solutions were refreshed every 12 h. Tests were performed in triplicate. The different toxicological end-points were observed under a stereomicroscope (Stereomicroscope, SMZ18, Nikon) (Nikon, Tokyo, Japan) at 4, 24, 48, 72, 96 and 120 hpf. Survival rate was analyzed by counting the number of live embryos or larvae at each time point. Heart rate and frequency of movements were recorded with a CCD camera attached to the stereomicroscope and the number of heartbeats and movements were counted by hand. All animal experiments were performed in full compliance with the revised directive 2010/63/EU.

#### 3.3.3. Data Analysis

Data were expressed as mean ±S.D. Statistic evaluation between each treatment group was calculated as previously described [38].

## Supplementary Materials

The following are available online at www.mdpi.com/2079-4991/7/12/414/s1.
