# Supplementary material for: Toxicity Assessment of Carbon Nanomaterials in Zebrafish during Development
Source: Nanomaterials (Basel). 2017 Nov 25;7(12):414. doi: 10.3390/nano7120414 (PMC5746904; doi:10.3390/nano7120414)
Supplement: Supplementary file 1 [file nanomaterials-07-00414-s001.docx]

Supplementary Materials

Toxicity Assessment of Carbon Nanomaterials in Zebrafish during Development

Marta d’Amora ^1^, Adalberto Camisasca ^1,2^, Stefania Lettieri ^1^ and Silvia Giordani ^1,3,^*

^1^ Nano Carbon Materials, Istituto Italiano di Tecnologia (IIT), via Livorno 60, 10144 Torino, Italy; marta.damora@iit.it (M.d.A.); adalberto.camisasca@iit.it (A.C.); stefania.lettieri@iit.it (S.L.)

^2^ Department of Chemistry and Industrial Chemistry, University of Genoa, via Dodecaneso 31, 16145
Genoa, Italy

^3^ Department of Chemistry, University of Turin, via Giuria 7, 10125 Turin, Italy

***** Correspondence: silvia.giordani@iit.it; Tel.: +39-0110917646

Received: 21 October 2017; Accepted: 22 November 2017; Published: 25 November 2017


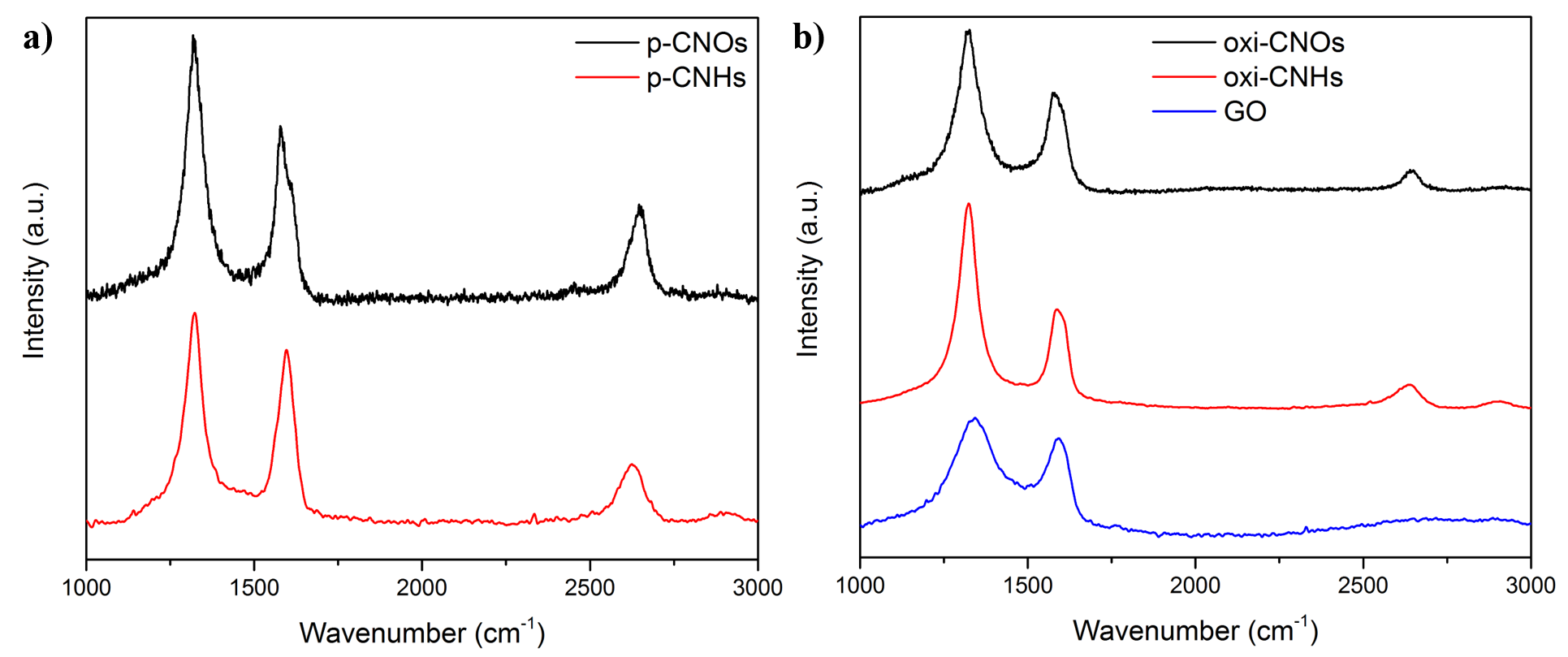


**Figure S1.** Raman spectra of (**a**) pristine CNOs (p-CNOs) (black) and pristine nanohorns (p-CNHs) (red); (**b**) oxidized carbon nano-onions (oxi-CNOs) (black), oxi-CNHs (red) and GO (blue). The Raman spectra are normalized to the G-band.

**Table S1**. I_D_/I_G_ ratio for the different carbon nano-materials.

|  | **p-CNOs** | **oxi-CNOs** | **p-CNHs** | **oxi-CNHs** | **GO** |
| --- | --- | --- | --- | --- | --- |
| **I_D_/I_G_** | 1.5 | 1.63 | 1.21 | 2.06 | 1.2 |


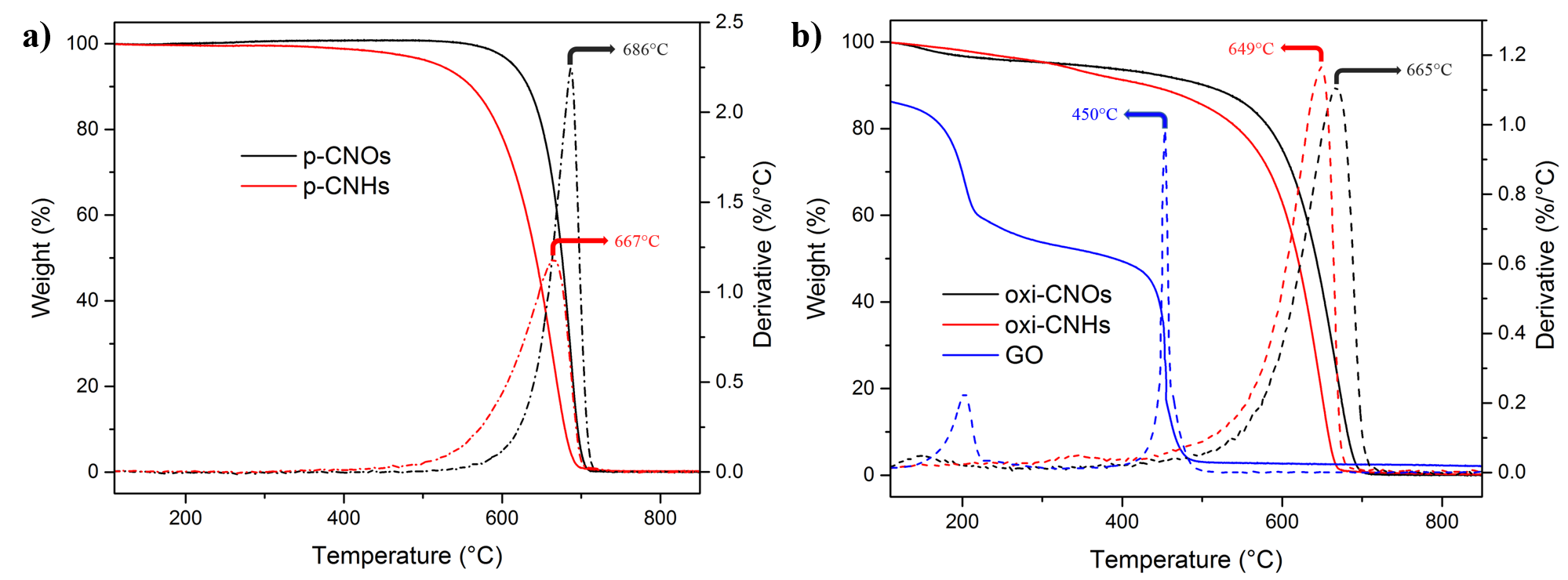


**Figure S2**. TGA (solid lines) and the corresponding weight loss derivatives (dotted lines) of (**a**) p-CNOs (black) and p-CNHs (red); (**b**) oxi-CNOs (black), oxi-CNHs (red) and GO (blue).

**Table S2**. Decomposition temperature for the different carbon nano-materials.

|  | **p-CNOs** | **oxi-CNOs** | **p-CNHs** | **oxi-CNHs** | **GO** |
| --- | --- | --- | --- | --- | --- |
| $\boldsymbol{T}_{\boldsymbol{d}}$ | 686°C | 665°C | 667°C | 649°C | 450°C |


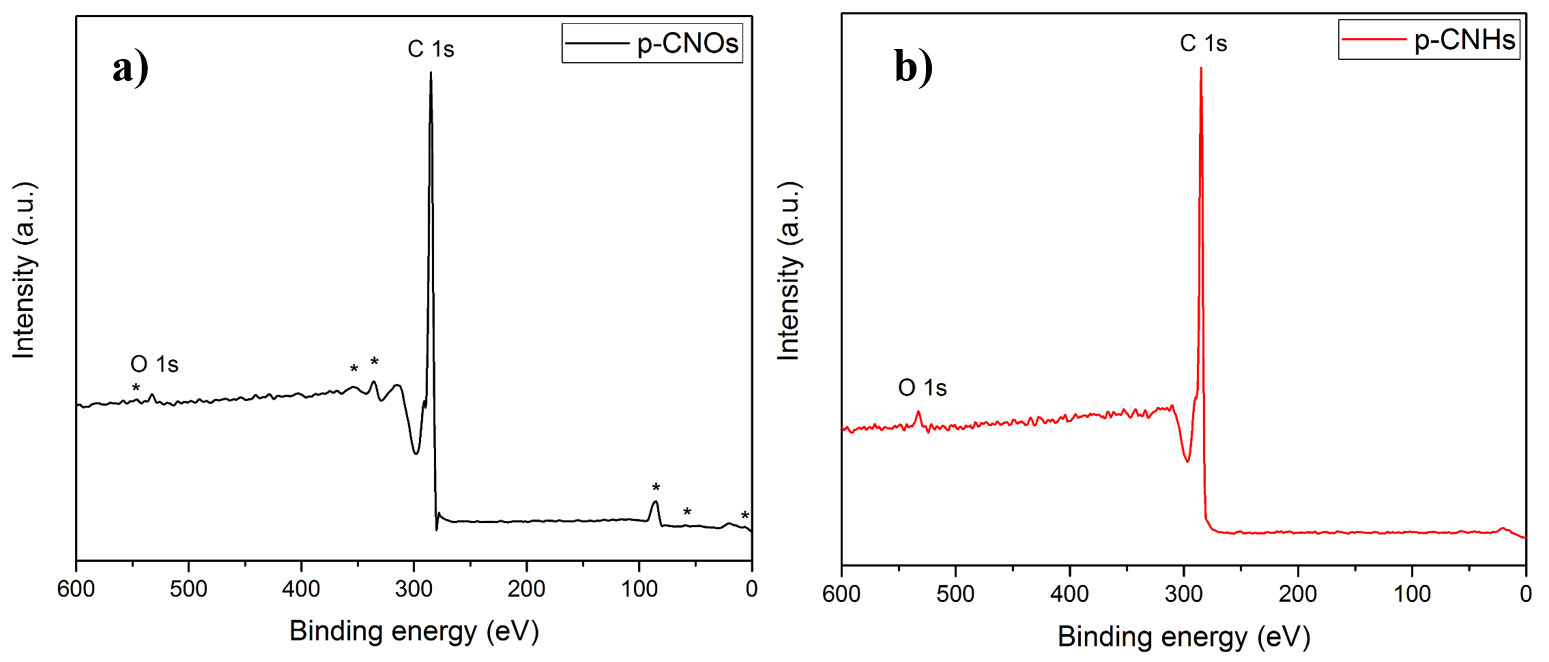


**Figure S3.** XPS survey spectra of (**a**) p-CNOs and (**b**) p-CNHs.


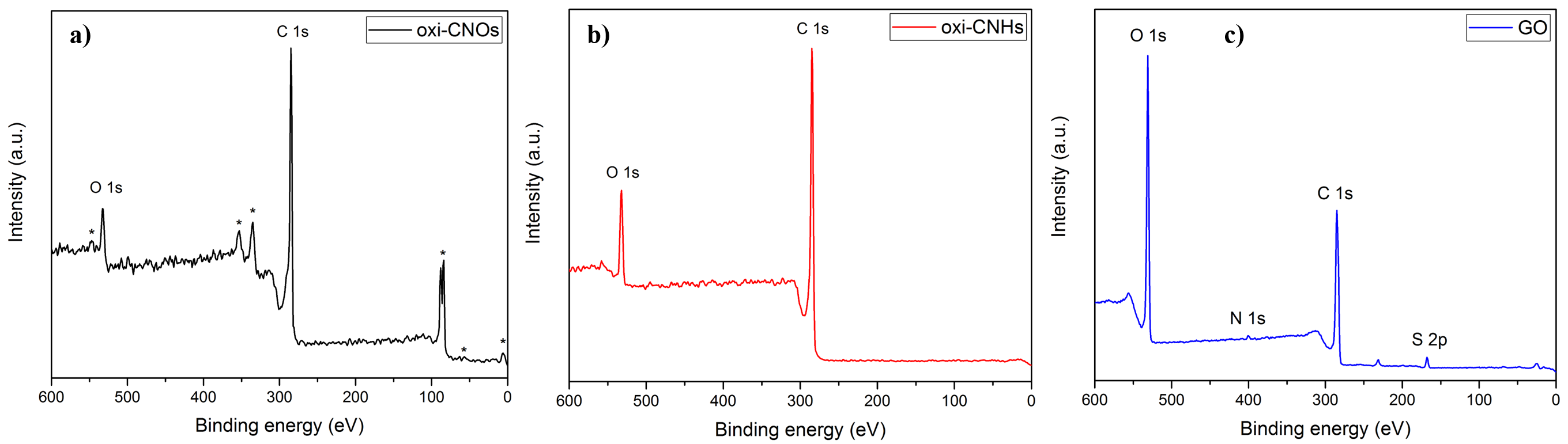


**Figure S4.** XPS survey spectra of (**a**) oxi-CNOs; (**b**) oxi-CNHs and c) GO.

**Table S3.** Atomic percentages from XPS analyses for p- and oxi-CNOs, p- and oxi-CNHs and GO.

| **Sample** | **C_1s_ (%)** | **O_1s_ (%)** | **N_1s_ (%)** | **S_2p_ (%)** |
| --- | --- | --- | --- | --- |
| p-CNOs | 99.00 | 1.00 | - | - |
| oxi-CNOs | 90.70 | 9.30 | - | - |
| p-CNHs | 98.00 | 2.00 | - | - |
| oxi-CNHs | 87.90 | 12.10 | - | - |
| GO | 69.73 | 27.66 | 2.06 | 0.56 |


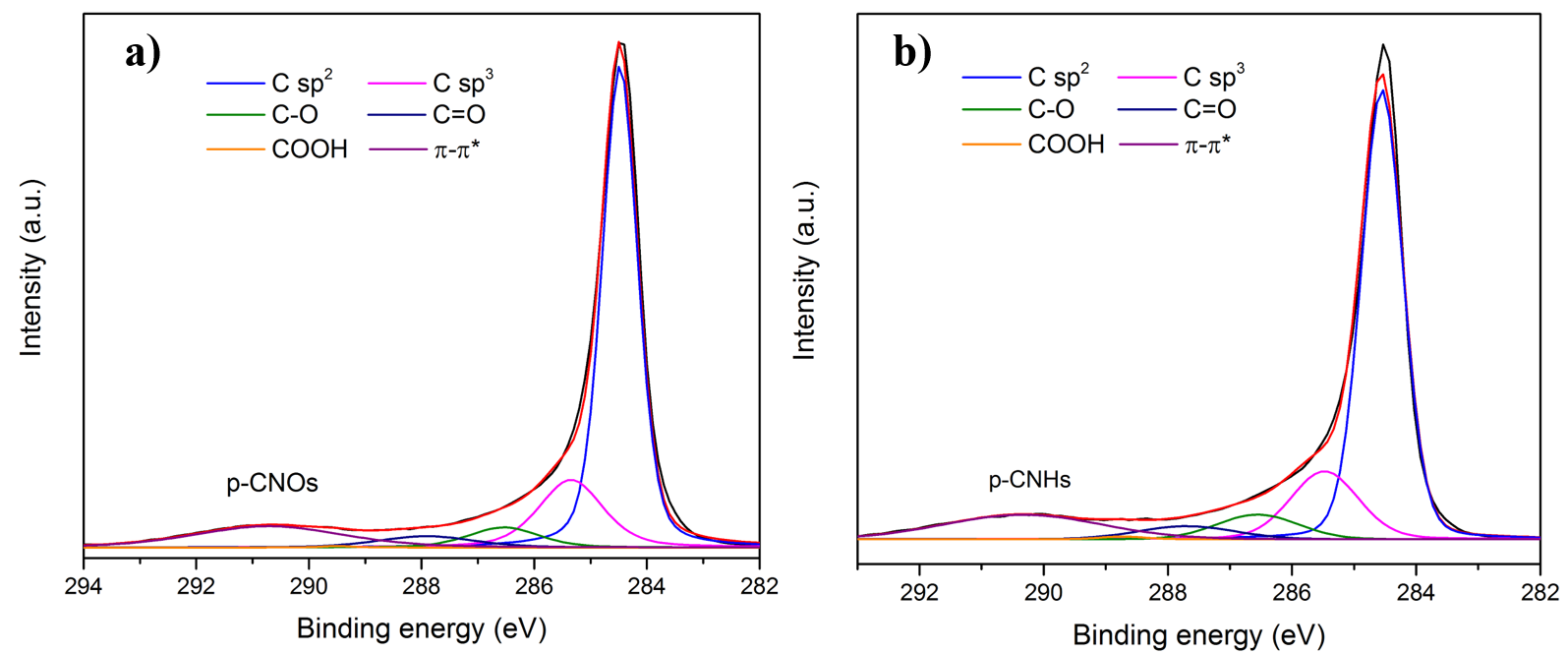


**Figure S5.** High-resolution XPS spectra of the C1s region of (**a**) p-CNOs and (**b**) p-CNHs, including peak-fitting analysis.

**Table S4.** Positions and the relative area percentages of the deconvoluted C1s peaks obtained from XPS analyses of p- and oxi-CNOs, p- and oxi-CNHs and GO.

| **Sample** | **C-C sp^2^ (eV)** | **C-C sp^3^ (eV)** | **C-O (eV)** | **C=O (eV)** | **COOH (eV)** | **π-π* (eV)** |
| --- | --- | --- | --- | --- | --- | --- |
| p-CNOs | 284.48 (62.66 %) | 285.35 (16.47 %) | 286.54 (5.17 %) | 287.92 (3.99 %) | 289.50 (0.18 %) | 290.71 (11.57 %) |
| oxi-CNOs | 284.48 (49.03 %) | 285.13 (30.25 %) | 286.65 (4.43 %) | 287.62 (1.95 %) | 289.06 (9.93 %) | 291.50 (4.40 %) |
| p-CNHs | 284.55 (62.59 %) | 285.48 (15.21 %) | 286.55 (6.15 %) | 287.69 (3.87 %) | 288.73 (0.35 %) | 290.34 (11.84 %) |
| oxi-CNHs | 284.45 (50.41 %) | 285.13 (35.32 %) | 286.61 (2.40 %) | 287.20 (1.24 %) | 288.63 (9.17 %) | 291.09 (1.46 %) |
| GO | 284.45 (40.84 %) | - | 286.48 (48.88 %) | 287.51 (7.61 %) | 288.69 (2.67 %) | 291.00 (0.00 %) |


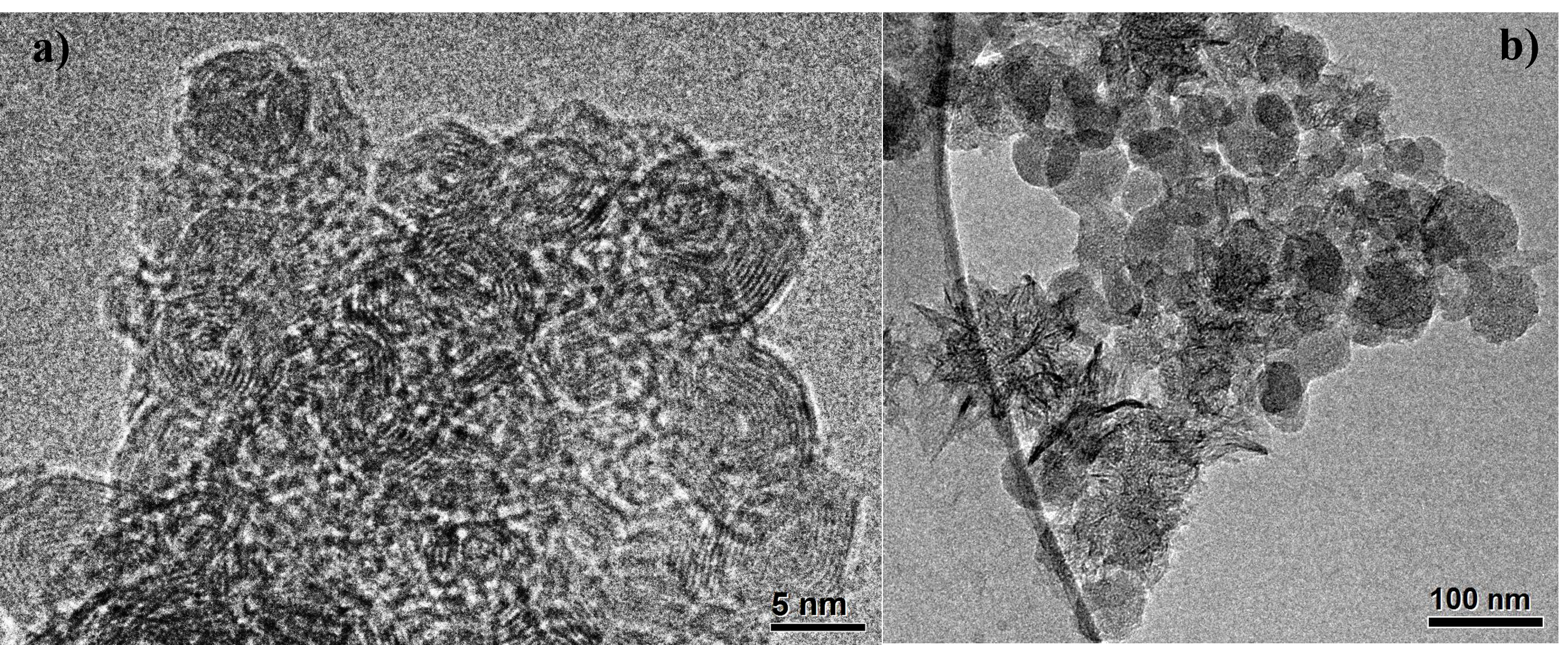


**Figure S6.** HRTEM images of (**a**) p-CNOs and (**b**) p-CNHs.

© 2017 by the authors. Licensee MDPI, Basel, Switzerland. This article is an open access

article distributed under the terms and conditions of the Creative Commons Attribution

(CC BY) license (http://creativecommons.org/licenses/by/4.0/).
